# Rural–Urban Differences in the Association Between Reproductive Coercion and Postpartum Family Planning

**DOI:** 10.1111/sifp.70050

**Published:** 2026-04-04

**Authors:** Jessica L. Dozier, Shannon N. Wood, Robel Yirgu, Solomon Shiferaw, Linnea A. Zimmerman

## Abstract

Access to timely postpartum family planning (PPFP) helps safeguard women's reproductive autonomy and supports healthy birth spacing, yet little is known about how reproductive coercion (RC) shapes women's ability to initiate contraception after childbirth. We analyzed prospective cohort data from 1,481 pregnant Ethiopian women followed for 12 months postpartum between 2021 and 2023. Time (in months) to contraceptive uptake by pre‐pregnancy RC exposure and residence was assessed using Kaplan–Meier estimators and parametric survival models. Approximately one in seven women experienced pre‐pregnancy RC, and, overall, 46.7 percent adopted a modern contraceptive method within 12 months postpartum. Overall differences in PPFP uptake were modest, but residence significantly modified this relationship. Rural women who experienced RC initiated postpartum contraception later and had a 40 percent lower hazard of initiating postpartum contraception compared to unexposed rural women (adjusted hazard ratio: 0.60, 95 percent confidence interval 0.37–0.98), while no significant association was observed among urban women. Urban women initiated PPFP more rapidly than rural women, regardless of RC exposure. These findings suggest that the effects of RC extend beyond pregnancy and are shaped by the wider structural context, particularly in rural settings where access to contraception may be limited. Recognizing RC as part of the PPFP context is essential for designing programs and health systems responses that support women to realize their reproductive goals and address interpersonal and structural barriers to timely contraceptive use.

## BACKGROUND

Access to timely initiation of postpartum family planning (PPFP), defined as starting contraception within 12 months of childbirth, is both a critical public health strategy and an essential component of women's reproductive rights (World Health Organization 2013). Short interpregnancy intervals (less than 24 months) are associated with elevated risks of maternal complications, preterm birth, low birthweight, and neonatal mortality (Ali, Bellizzi, and Shah 2023; Conde‐Agudelo et al. 2012; Yaya et al. 2020). Despite strong desire among many postpartum women in low‐ and middle‐income countries to avoid or delay subsequent pregnancies for at least two years after giving birth, actual uptake of PPFP remains low (Dev et al. 2019; Yemane et al. 2021). Moreover, many women experience substantial delays in initiating contraception, which increases their vulnerability to adverse health outcomes for both mother and child (World Health Organization 2013; Dev et al. 2019; Yemane et al. 2021). Barriers to timely PPFP initiation include limited access to services, particularly in rural areas, restrictive sociocultural norms, and unsupportive partners (Dev et al. 2019; Hounton et al. 2015; Kriel et al. 2019; Yemane et al. 2021). In sub‐Saharan Africa, patriarchal norms and inequitable power dynamics often facilitate men to have greater control over decisions concerning contraceptive use, pregnancy timing, and family size; and, as a result, women frequently have shorter birth intervals than they desired (Karp et al. 2020; Rutstein 2011).

Behaviors such as pregnancy coercion (e.g., where a partner pressures a woman to become pregnant or not use contraception), birth control sabotage (e.g., tampering with or hiding contraceptives), and control over pregnancy outcomes (e.g., attempts to force decisions regarding continuing or terminating a pregnancy) all constitute reproductive coercion (RC) (Silverman and Raj 2014; Grace and Fleming 2016). This form of intimate partner violence directly violates women's reproductive autonomy and their right to make reproductive choices free from coercion, manipulation, threats, or violence (Silverman and Raj 2014; United Nations and The Office of the High Commissioner for Human Rights 1993). Population‐based studies in sub‐Saharan Africa estimate that up to one in five women of reproductive age experience RC within a given year (Wood et al. 2023). Reproductive Coercion has been linked to reduced contraceptive use, increased rates of covert contraceptive behavior, and increased risk of unintended pregnancy (Grace and Fleming 2016; Wood, Dozier, et al. 2022; Silverman et al. 2019, 2020; Dozier et al. 2022). Furthermore, research points to a cyclical pattern in which RC may prompt women to use contraception covertly, which, when discovered, can escalate partner control or violence (Dozier et al. 2022; Wood, Kennedy, et al. 2022). Recent national evidence from Ethiopia suggests that RC is associated with a higher likelihood of women experiencing very short intervals between pregnancies (less than 12 months) (Dozier, Wood, et al. 2025), underscoring the serious reproductive health consequences of partner control. While these findings point to RC as a driver of rapid repeat pregnancies, the question about whether RC contributes to *delayed* postpartum contraceptive uptake remains unanswered.

Understanding when women initiate—or do not initiate—PPFP helps ensure that interventions that reach them at the right time, particularly given the heightened motivation to avoid pregnancy and increased contact with the health system through antenatal care, postnatal care, and routine childhood immunization services (Moore et al. 2015; USAID 2017; World Health Organization 2013). Each of these encounters represents a critical opportunity to strengthen PPFP counseling and support, ensuring that women's needs are met during the postpartum period and beyond (World Health Organization 2013). Monitoring contraceptive use trends over the first year postpartum can help identify where such interventions may be most impactful (World Health Organization 2013; Moore et al. 2015). Similarly, insights into the relationship between RC and postpartum contraceptive behavior can guide when and how to integrate prevention and harm‐reduction strategies into routine maternal and child health services, with the goal of ensuring women have support and options to use contraception when and if they choose.

Ethiopia provides a compelling context for examining the relationship between RC and PPFP uptake. The country's persistently high fertility rates, strong sociocultural norms favoring large families and early childbearing, and regional disparities in family planning services all contribute to unmet need for PPFP (Chuta, Birhanu, and Vinci 2020; Kapadia‐Kundu et al. 2022; Karp et al. 2020; McClendon et al. 2018). The majority of postpartum women in Ethiopia report wanting to avoid becoming pregnant or delay another pregnancy by at least two years (Chuta, Birhanu, and Vinci 2020; McClendon et al. 2018); however, less than half begin using a modern contraceptive method within 12 months of delivery (Addis Ababa University School of Public Health and The Bill & Melinda Gates Institute for Population and Reproductive Health at The Johns Hopkins Bloomberg School of Public Health 2022) with persistent delays, especially prevalent in rural areas (Dona et al. 2018). Furthermore, up to one in five Ethiopian women of reproductive age reports experiencing RC in the past year (Wood, Dozier, et al. 2022), and one in three experiences RC in the year before pregnancy (Dozier, Zimmerman, et al. 2025). Yet, no study in Ethiopia or elsewhere has examined whether women who experience RC face delays in initiating postpartum contraception compared to those who do not experience RC.

In addition, Ethiopia is also well‐suited to investigate whether urban or rural residence moderates the relationship between RC and PPFP uptake. Marked differences in health infrastructure, sociocultural norms, and gender equity influence both contraceptive access and reproductive autonomy. Delays in postpartum contraceptive initiation are especially pronounced in rural areas, where access to services is more limited, and patriarchal norms may be stronger, posing distinct barriers for women who wish to use contraception (Karp et al. 2020; Central Statistical Agency and ICF 2017; Addis Ababa University School of Public Health and The Bill & Melinda Gates Institute for Population and Reproductive Health at The Johns Hopkins Bloomberg School of Public Health 2022).

By leveraging longitudinal data from a cohort of pregnant Ethiopian women, this study examines whether RC is associated with delayed postpartum uptake and explores whether these associations differ by rural versus urban residence. We hypothesized that women who experienced RC before pregnancy would be less likely to initiate PPFP and do so more slowly than women who did not experience RC. We further expected these effects to be more pronounced in rural areas.

## METHODS

### Data Source and Study Design

We conducted a secondary analysis of data from the Performance Monitoring for Action Ethiopia (PMA Ethiopia) study (2021–2023), a regionally representative prospective cohort study of pregnant and recently postpartum women followed through 12 months postpartum. Data were collected by trained local interviewers using mobile technology between November 2021 and September 2023 across four regions that collectively represent 70 percent of Ethiopia's total population: Addis Ababa, Amhara, Oromia, and the Southern Nations, Nationalities, and Peoples’ Region (SNNP).^1^ Researchers from Addis Ababa University and Johns Hopkins University designed, implemented, analyzed, and wrote up this study. The design and implementation details have been described elsewhere (Zimmerman et al. 2020).

Briefly, PMA Ethiopia employed a multistage stratified survey design, selecting 162 clusters (enumeration areas) across the study regions, with probability proportional to size, and stratified by urban and rural areas in Amhara, Oromia, and SNNP. A household census identified women aged 15–49. Of 25,183 women screened, 2,306 were eligible (i.e., currently pregnant or less than six weeks postpartum) and 2,261 completed the baseline interview. Women who were over six weeks postpartum at baseline were excluded (*n* = 3), resulting in a cohort of 2,258 women. Follow‐up interviews occurred at approximately six weeks, six months, and one year postpartum. Overall, 2,111 (93.5 percent) women completed at least one follow‐up interview.

### Measures

#### Outcome Variables

Our primary outcome was time (in months) to uptake of a modern contraceptive method during the 12 months after childbirth. Descriptive analyses of contraceptive discontinuation are presented in the Supporting Information (Table S1). Modern methods include male or female sterilization, implants, intrauterine devices, injectables, pills, male or female condoms, emergency contraception, and lactational amenorrhea. We used a 12‐month retrospective calendar collected at follow‐up interviews to identify the first month postpartum when contraception was initiated. The calendar captured month‐by‐month reproductive events, including contraceptive use, pregnancy, birth, and abortion, since the end of the index pregnancy (i.e., the pregnancy for which women were enrolled). At six‐month and one‐year interviews, women reported events for months 1–6 and 7–12, respectively. For women missing the six‐month interview, a full 12‐month history was collected at one year.

As in previous studies using reproductive calendars (Anglewicz et al., 2023; Tsui et al., 2021), we minimized recall bias by prioritizing cross‐sectional reports of contraceptive use at each follow‐up when they conflicted with calendar data. For example, if a woman reported initiating contraception immediately postpartum at the six‐week interview, but reported later initiation at the six‐month interview, the initial report was used.

#### Exposure Variable

Our exposure of interest was male partner‐perpetrated RC occurring in the 12 months before the baseline interview, which took place during pregnancy. Women reported RC experiences using five items adapted from the pregnancy coercion subscale of the Reproductive Coercion Scale (RCS), which was developed in the United States (McCauley et al. 2017) and later validated among samples of reproductive‐age women in sub‐Saharan Africa, including Ethiopia (Wood et al. 2023; Wood, Dozier, et al. 2022). In PMA Ethiopia, minor wording changes, such as substituting “birth control” with “family planning” to reflect local terminology, were made. Additionally, one original RCS item (“told you not to use any birth control”) was divided into two separate questions (“made you feel bad or treated you badly for wanting to use a family planning method to delay or prevent pregnancy” and “tried to force or pressure you to become pregnant”), an adaptation based on formative work in Niger (Grace and Fleming 2016; Wood, Dozier, et al. 2022; Silverman et al. 2019, 2020) and used in other PMA surveys (Dozier et al. 2022; Wood et al. 2023). At baseline, pregnant women were asked: in the past 12 months has your husband/partner (1) made you feel bad or treated you badly for wanting to use a family planning method to delay or prevent pregnancy; (2) tried to force or pressure you to become pregnant; (3) said he would leave you if you didn't get pregnant, (4) told you he would have a baby with someone else if she didn't get pregnant; (5) taken away your family planning or kept you from going to the clinic to get family planning. RC was defined as any affirmative response to any item (Cronbach's alpha = 0.81 in the present sample). Because women are less likely to face pressure to become pregnant once they are pregnant, we included gestational age at baseline in our analysis to account for variable time at risk for RC.

#### Covariates

Covariates included in the adjusted models were residence (urban/rural), polygynous union status (yes/no), women's highest level of education (never attended, primary school, secondary or higher), parity (inclusive of index pregnancy, classified as 1, 2–3, 4 or more), ever use of family planning (yes/no), resumption of sexual activity during the postpartum period (coded as a monthly time‐varying yes/no variable for each of the 12 postpartum months), and gestational age of index pregnancy at baseline (1–9 months). Residence, polygyny, education, ever use of family planning, and gestational age were measured at baseline. No covariate data were missing. Other variables considered, but not included in adjusted models, were region, women's age, religion, partner's age, and partner's education. These variables were used to describe the sample.

### Analytic Sample

To ensure a clear temporal ordering of RC, index pregnancy, and PPFP, women who delivered before the baseline interview were excluded from analysis (*n* = 487), as well as those not partnered at baseline (*n* = 37), missing postpartum follow‐up (*n* = 121 excluded), missing RC data (*n* = 1), or with non‐live birth outcomes (stillbirth, miscarriage, or abortion; *n* = 131). The final analytic sample was *n* = 1,481.

### Statistical Analysis

Analyses accounted for complex survey design, including multistage cluster sampling, stratification, and sample weights that reflected the differential probability of selection of enumeration areas and loss‐to‐follow‐up. Loss‐to‐follow‐up did not differ by RC exposure. We first described weighted distributions of sociodemographic characteristics, RC, and PPFP adoption. Descriptive analyses of discontinuation by RC exposure, limited by the small number of events, are presented in Table S1 in the Supporting Information.

Kaplan–Meier survival analysis estimated the time to contraceptive adoption, comparing women with and without RC exposure. We produced two failure curves: one overall curve and the other curve stratified by urban or rural residence, and used Wilcoxon tests to assess the differences. Women were right censored in the first month they reported pregnancy or were lost to follow‐up.

Due to violation of proportional hazards assumptions, which was assessed via Schoenfeld residuals, we used parametric survival regression with a Weibull distribution to model time to contraceptive uptake, allowing hazards to vary across time. Covariates with *p* < 0.2 in bivariate models (not shown) were considered for inclusion in multivariable models; due to collinearity with parity, age was excluded.

To examine effect modification by residence, an interaction term between RC and urban/rural residence was included. Significant interaction was present and retained in the final model (Model 2). All analyses were conducted using Stata version 16.1 (StataCorp 2019).

### Ethical Considerations

Oral informed consent was obtained for all participants. Trained interviewers administered the full informed consent at study baseline and reaffirmed consent to continue participation at each subsequent follow‐up interview. Data collection adhered to the best practices for research on violence against women (World Health Organization 2016). This included a universal screening for distress and referral to health centers for violence and reproductive health support, regardless of violence disclosure. Institutional Review Boards at Addis Ababa University and Johns Hopkins Bloomberg School of Public Health approved all procedures.

## RESULTS

The majority of women (74.1 percent) resided in rural areas, primarily from Oromia (52.7 percent), followed by Amhara (21.5 percent), SNNP (21.0 percent), and Addis Ababa (4.8 percent) (Table 1). Over half were aged 25–34 (54.0 percent), and nearly half had four or more children (45.5 percent). Almost half (46.0 percent) had attained a primary education. Religious affiliation was nearly evenly distributed among Muslims (36.2 percent), Orthodox Christians (33.2 percent), and Protestants (28.3 percent). Notably, over one‐third (35.0 percent) reported never having used contraception, and a large majority (91.4 percent) had resumed sexual activity since giving birth. Partners were predominantly under 35 years old (57.4 percent). About one‐third of male partners had a secondary education or higher, compared to about one‐fourth of women.

**TABLE 1:** Sample characteristics

Characteristics^a^	Weighted (%)	Weighted (*n*)	Unweighted (*n*)
Region			
Addis Ababa	4.8	72	214
Amhara	21.5	318	331
Oromiya	52.7	781	531
SNNP	21.0	310	405
Residence			
Urban	25.9	384	647
Rural	74.1	1097	834
Women's age			
15–24	12.7	188	147
25–34	25.5	378	372
25–29	28.5	422	453
30–34	19.4	288	296
35–39	10.8	160	173
40–49	3.0	45	40
Parity^b^			
One child	16.7	247	267
Two to three children	37.8	560	610
Four children or more	45.5	674	604
Women's education			
None	29.9	443	379
Primary	46.0	681	641
Secondary or higher	24.1	357	461
Religion			
Orthodox	33.2	420	442
Muslim	36.2	535	582
Protestant	28.3	420	435
Other	2.3	34	22
Gestational age, mean (SD)	5.2 (2.1)		
Ever used a method of contraception			
Yes	65.0	963	1010
No	35.0	518	471
Polygynous relationship			
No	92.2	1366	1485
Yes	7.8	115	96
Partner's age			
18–24	9.4	140	109
25–34	48.0	711	737
35–44	30.7	455	472
≥45	11.9	176	163
Partner's education			
None	27.9	414	337
Primary	41.1	609	566
Secondary or higher	31.0	459	578
Resumed sex^c^			
Yes	91.4	1353	1351
No	8.6	127	130
Total	100	1481	1481

^a^Unless otherwise noted, sample characteristics were measured during enrollment (i.e., during pregnancy).

^b^Inclusive of index pregnancy.

^c^Measured during the postpartum period.

About one in seven (13.8 percent) women reported any RC in the 12 months before the index pregnancy (Table 2), with RC more common in rural than urban areas (15.1 percent vs. 10.2 percent; *p* = 0.02; Table S2 in the Supporting Information). Among women who experienced RC, 40.0 percent adopted PPFP, compared to 47.6 percent of women who did not experience RC (*p* = 0.11).

**TABLE 2:** Postpartum Contraceptive use by RC exposure

			Reproductive coercion, *n* (%)		
	Weighted n (%)	Unweighted *n*	Yes	No	*p*
Overall	1481 (100)	1481	205 (13.8)	1276 (86.2)	
Contraceptive Use					0.11
Yes	692 (46.7)	769	82 (40.0)	610 (47.8)	
No	789 (53.3)	712	123 (60.0)	666 (52.2)	

**FIGURE 1:**
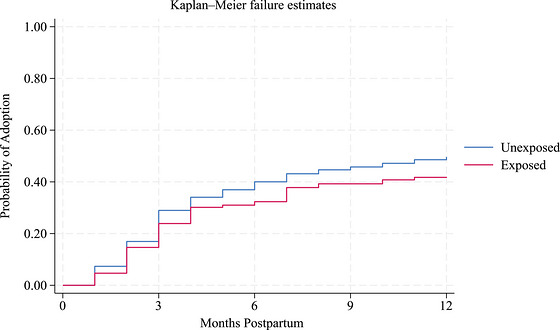
Failure curves for postpartum contraceptive uptake by reproductive coercion (RC). Differences between RC‐exposed and unexposed were assessed using the Wilcoxon test, *p* = 0.15; no statistically significant difference in uptake timing was observed between the two groups

Figure 1 shows Kaplan–Meier failure curves estimating cumulative PPFP adoption by RC exposure. At 3, 6, and 12 months postpartum, the probability of contraceptive adoption was consistently higher among unexposed women (29.0 percent, 40.0 percent, 49.6 percent) compared to those exposed to RC (23.9 percent, 32.3 percent, 41.9 percent), but the results were not statistically significant (*p* = 0.15)

**FIGURE 2:**
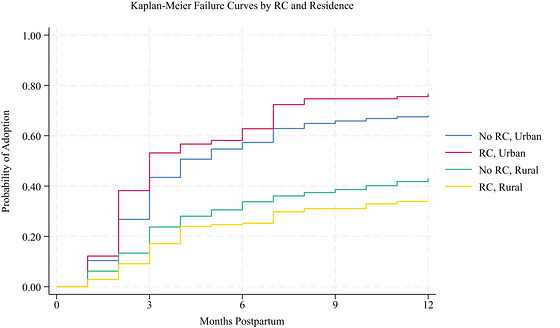
Failure curves for postpartum contraceptive uptake by reproductive coercion (RC) exposure and residence. The global test indicated statistically significant differences in postpartum contraceptive uptake by RC exposure and residence (*p* < 0.001), while the pairwise comparison between RC‐exposed and unexposed women in rural groups was marginally significant (*p* = 0.08); no significant difference was observed in urban groups (*p* = 0.18). Among women unexposed to RC, uptake occurred significantly earlier in urban compared to rural settings (Wilcoxon test, *p* < 0.001). Among RC‐exposed women, urban residents also initiated uptake substantially earlier than rural residents (Wilcoxon test, *p* < 0.001)

Stratification by RC and residence revealed greater and statistically significant differences (Figure 2). Among rural women, those who were exposed to pre‐pregnancy RC initiated PPFP marginally faster than rural women who did not experience RC (*p* = 0.08). By 3, 6, 12 months postpartum, 17.1 percent, 25.2 percent, and 33.9 percent of rural women who experienced RC had initiated PPFP, compared to 23.7 percent, 33.8 percent, and 43.1 percent of rural women who did not experience RC. There were no differences in uptake among urban women who did and did not experience pre‐pregnancy RC (*p* = 0.18). Urban women adopted PPFP faster than their rural counterparts, regardless of RC exposure; among women who experienced pre‐pregnancy RC, urban women initiated contraception faster than their rural counterparts (*p* < 0.001).

Unadjusted survival models (Table 3) indicated a lower hazard of PPFP adoption among women exposed to RC, but this result was only marginally significant (hazard ratio (HR): 0.79, 95 percent confidence interval [CI]: 0.60–1.04, *p* = 0.09). Adjusted results without interaction (Model 1) showed no significant overall association between RC and PPFP adoption (adjusted HR: 0.91, 95 percent CI: 0.70–1.18, *p* = 0.46). Polygyny, higher parity, and lower education were linked to reduced hazards of PPFP uptake, while prior contraceptive use and resuming sexual activity were associated with increased hazards of uptake.

**TABLE 3:** Hazard of adoption of modern contraception within one year of delivery, by experience of reproductive coercion (*n* = 1,481)

	Crude model	Adjusted model 1	Adjusted model 2
	HR (95% CI)	aHR (95% CI)	aHR (95% CI)
Any RC (ref: no)			
	0.79 (0.61–1.04)	0.91 (0.70‐1.18)	1.29 (0.92–1.83)
	p = 0.09	p = 0.46	p = 0.14
Area of residence (ref: urban)			
Rural	0.43 (0.36–0.50) ^***^	0.68 (0.56–0.83) ^***^	0.72 (0.59–0.88) ^***^
Interaction term between RC and residence	‐	‐	0.60 (0.37–0.98) ^*^
Partner has other wives (ref: no)			
Yes	0.43 (0.28–0.66)^***^	0.71 (0.47–1.08)	0.72 (0.47–1.10)
**Parity ^1^** (ref: 1 child)			
Two to three children	0.61 (0.48–0.77) ^***^	0.49 (0.39–0.62)^***^	0.50 (0.39–0.62) ^***^
Four children or more	0.29 (0.23–0.38) ^***^	0.34 (0.26–0.45)^***^	0.35 (0.26–0.46)^***^
Education (ref: never attended)			
Primary	1.78 (1.40–2.26) ^***^	1.39 (1.07–1.81) ^*^	1.40 (1.08–1.82) ^*^
Secondary or higher	3.29 (2.58–4.21) ^***^	1.54 (1.14–2.09) ^**^	1.57 (1.16–2.11) ^**^
Ever used a method of contraception (ref: no)			
Yes	3.12 (2.48–3.91) ^***^	2.91 (2.31–3.68) ^***^	2.90 (2.29–3.61) ^***^
Sexual activity (ref: not resumed)	2.46 (1.57–3.85) ^***^	2.30 (1.46–3.63) ^***^	2.29 (1.46–3.61) ^***^
Gestational age (at baseline)	1.01 (0.97–1.05)	1.05 (1.00–1.09) ^*^	1.05 (1.01–1.09)^*^

NOTE: Crude RC models include gestational age at baseline. aHR, adjusted hazards ratio; CI, confidence interval; RC, reproductive coercion.

* *p* < 0.05; ** *p* < 0.01; *** *p* < 0.001.

Introducing an interaction by residence (Model 2) revealed a significant effect modification. Among rural women, experiencing RC significantly reduced the hazard of PPFP adoption by 40 percent (interaction adjusted hazard ratio [aHR]: 0.60, 95 percent CI 0.37–0.98, *p* < 0.05). Among unexposed women, rural residence was associated with a 28 percent lower hazard of adoption compared to urban areas (aHR: 0.72, 95 percent CI: 0.59–0.88; *p* < 0.01). Among urban women, RC was associated with a nonsignificant increased hazard of PPFP adoption (aHR: 1.29, 95 percent CI: 0.92–1.83, *p* = 0.14). Other covariate associations were consistent across models.

## DISCUSSION

This population‐based prospective study is the first to examine how RC shapes the timing of postpartum contraceptive uptake, identifying RC as a previously unexamined determinant of PPFP in Ethiopia. We found that women living in rural areas who experienced RC in the year preceding pregnancy initiated PPFP less frequently and significantly later than their counterparts who had not experienced RC, even after adjusting for sociodemographic and reproductive factors. These findings suggest that the effects of RC extend beyond pregnancy itself and, in rural settings, continue to shape women's ability to use PPFP. By leveraging longitudinal data, our study provides novel evidence suggesting that RC constrains reproductive autonomy beyond pregnancy and interacts with wider structural inequities in the postpartum period, particularly in rural settings where access to contraception may be limited.

Our findings align with prior research identifying parity, education, and reproductive history as key determinants of postpartum contraceptive behavior (Dev et al. 2019; Hounton et al. 2015; Yemane et al. 2021) while extending this literature by demonstrating that pre‐pregnancy RC independently influenced the timing of PPFP initiation among rural women. One likely explanation is that RC reflects entrenched power imbalances in heterosexual relationships that do not end with childbirth. Without targeted efforts to address these dynamics, controlling behaviors may persist into the postpartum period, as documented in qualitative work from sub‐Saharan Africa (Boyce et al. 2020; Thomas et al. 2024). Women who have previously experienced RC may anticipate conflict, retaliation, or other negative partner responses if they attempt to use PPFP (Thomas et al. 2024). Taken together, these findings emphasize the importance of recognizing the personal risks some women face when they are considering PPFP (World Health Organization 2013) and underscore the need for family planning programs to be attentive to the wider relationship dynamics. For couples where partner involvement is safe and welcomed by women, educational efforts should foster respectful, shared decision‐making and promote gender equity in reproductive health (USAID 2018).

The observed differences in postpartum contraceptive initiation between rural and urban women highlight the influence of structural context on contraceptive behavior, particularly in the presence of RC. In rural settings, limited health infrastructure, fewer service delivery points, and reduced access to discreet or woman‐controlled methods can narrow women's contraceptive options (Alem and Agegnehu 2021; Kibira et al. 2020). These constraints are often compounded by stronger patriarchal and pronatalist norms, making it more difficult for women, particularly those with an unsupportive or abusive partner, to make autonomous reproductive decisions (Thomas et al. 2024; Kibira et al. 2020). In these settings, RC is more likely to translate into real delays or nonuse, as women have fewer ways to circumvent partner opposition (e.g., covert use, supportive networks). Delayed initiation, in turn, increases the risk of unintended and closely spaced pregnancies and points to the need for multi‐level interventions in rural areas that address not only individual behavior but also interpersonal and health‐system barriers.

Urban women, by contrast, appear to be better positioned for the initiation of postpartum contraception despite experiences of RC. Greater access to services, broader method choice, and more opportunities for discreet use may allow some women to find ways to obtain contraception even in the face of partner opposition (Teferi and Schröders 2023; Anyatonwu and San Sebastián 2022; Wadei et al.). This is consistent with qualitative research from sub‐Saharan Africa showing covert contraceptive use can be an important safety strategy for women seeking to space or limit pregnancies when partners are unsupportive (Karp et al. 2020; Kibira et al. 2020). While covert use is not a substitute for equitable decision‐making, urban contexts may offer women more flexibility and options, weakening the observable impact of RC on postpartum contraceptive behavior.

This study also adds to the growing body of evidence linking RC to reproductive health outcomes (Dozier et al. 2022; Wood, Dozier, et al. 2022; Dozier, Wood, et al. 2025; Silverman et al. 2019, 2020). Unlike most prior studies, which rely on cross‐sectional data, our prospective design establishes a clear temporal sequence between RC and subsequent contraceptive behavior. The findings suggest that delayed postpartum contraceptive initiation may be one pathway through which RC contributes to short interpregnancy intervals and unintended pregnancies documented in earlier research (Grace and Fleming 2016; Dozier, Wood, et al. 2025). Our results help clarify how partner interference before pregnancy can shape reproductive trajectories well into the postpartum period.

Findings should be interpreted in light of several limitations. RC was only measured for the 12 months preceding baseline and not during the postpartum period, limiting our ability to assess ongoing coercion after delivery. Still, the longitudinal strengthens confidence in the temporal ordering of exposure and outcome. Although regions from which the sample was drawn from cover over 70 percent of Ethiopia's population, the findings may not be generalizable to unrepresented regions where cultural practices around gender roles and family planning, as well as differences in health service access, including remote geography and humanitarian challenges, may influence RC and PPFP uptake. Finally, we cannot determine whether nonuse of PPFP reflects women's autonomous choice or constrained autonomy. From a rights‐based perspective, nonuse of PPFP may be appropriate when it aligns with women's preferences (Senderowicz 2020). At the same time, national data indicate that many postpartum women intend to use contraception and commonly cited reasons for nonuse include biological misperceptions, fears of side effects, or partner influence (Magalona et al. 2023).

The findings have important implications for programs and policy. Efforts to reduce unmet need for postpartum contraception in rural Ethiopia are unlikely to succeed if focusing solely on expanding supplies or improving counseling with addressing RC and the broader conditions that sustain it. Community‐based, gender transformative interventions that engage men have shown promise in shifting inequitable gender norms and reducing intimate partner violence in sub‐Saharan Africa, suggesting one possible avenue for addressing norms that legitimize partner control over fertility decisions (Jewkes et al. 2008; Doyle et al. 2018). Such efforts could include couple‐focused counseling and targeted education for male partners about PPFP (USAID 2018). In parallel, engaging men as allies should emphasize the promotion of supportive partnership norms and shared responsibility for maternal and child health while avoiding forms of involvement that reinforce control over women's reproductive decisions (USAID 2018; Silverman and Raj 2014).

Health systems also play a critical role. Interventions that address RC should prioritize women's safety and autonomy while equipping providers with tools to identify and respond to RC. This includes training in trauma‐informed, rights‐based care; creating opportunities for confidential counseling; and ensuring access to discreet, women‐controlled contraceptive methods. Models such as ARCHES (Addressing Reproductive Coercion in Health Settings), which support providers in recognizing RC, offering patient‐centered counseling, discussing harm‐reduction strategies, and linking women to local support services (Uysal et al. 2023; Miller et al. 2016), could be adapted for the Ethiopian context and integrated into antenatal, postnatal, and PPFP services.

Antenatal care may be a particularly important entry point. Nearly three‐quarters of Ethiopian women receive antenatal care from a skilled provider, (Addis Ababa University School of Public Health and The Bill & Melinda Gates Institute for Population and Reproductive Health at The Johns Hopkins Bloomberg School of Public Health 2022) creating opportunities to discuss short‐ and long‐term fertility intentions, identify signs of partner interference, provide confidential counseling, and plan for postpartum contraceptive in ways that align with women's goals. Strengthening the integration of PPFP counseling into antenatal care, alongside investments in rural health infrastructure and community‐based supports, may help create conditions in which women are better able to access contraception on their own terms.

## CONCLUSION

This study contributes to understanding PPFP in Ethiopia by showing that RC is an important and largely overlooked barrier to timely contraceptive uptake, particularly for women in rural settings. Using longitudinal data, we find that experiences of RC before pregnancy can continue to shape women's reproductive trajectories after childbirth, influencing when or whether they initiate contraception. These findings point to the need for health system and policy responses that recognize RC as part of the PPFP context and support the development and evaluation of approaches that address partner interference within antenatal and postpartum care.

## ETHICS STATEMENT

Institutional Review Boards at Addis Ababa University (Ref: AAUMF 01‐008) and the Johns Hopkins Bloomberg School of Public Health (FWA00000287) approved study procedures.

## Supporting information




**Table S1**. Postpartum Contraceptive use and Discontinuation among contraceptive users by RC exposure.
**Table S2**. Distribution of reproductive coercion experience by residence.

## Footnotes

1 The Southern Nations, Nationalities, and People's Region was divided into new regional states, starting in 2020. For simplicity, we report the administrative boundaries as they were when the PMA Ethiopia 2021–2023 cohort was conducted. Design weights were applied to generate representative estimates of the combined population of the study regions as of 2021. Together, these regions represent ∼70 percent of the country's population.
