# Supplementary material for: Rural–Urban Differences in the Association Between Reproductive Coercion and Postpartum Family Planning
Source: Stud Fam Plann. 2026 Apr 4;57(2):193–208. doi: 10.1111/sifp.70050 (PMC13275037; doi:10.1111/sifp.70050)
Supplement: Supplementary file 1 — Table S1. Postpartum Contraceptive use and Discontinuation among contraceptive users by RC exposure. Table S2. Distribution of reproductive coercion experience by residence. [file SIFP-57-193-s001.docx]

**Table S1.** Postpartum Contraceptive use and Discontinuation among contraceptive users by RC exposure

|  |  |  | Reproductive coercion | |  |
| --- | --- | --- | --- | --- | --- |
|  | Weighted  n (%) | Unweighted n | Yes | No | p |
|  |  | n (%) | | |  |
| Overall | 1481 (100) | 1481 | 205 (13.8) | 1276 (86.2) |  |
| Contraceptive Use |  |  |  |  | 0.11 |
| Yes | 692 (46.7) | 769 | 82 (40.0) | 610 (47.8) |  |
| No | 789 (53.3) | 712 | 123 (60.0) | 666 (52.2) |  |
| Discontinuation^1^ |  |  |  |  | 0.75 |
| Yes | 178 (23.1) | 182 | 22 (24.5) | 156 (22.9) |  |
| No | 591 (76.9) | 587 | 69 (75.6) | 522 (77.1) |  |

^1^ among postpartum contraceptive users, n=769

**Table S2.** Distribution of reproductive coercion experience by residence

|  |  | Reproductive coercion | |  |
| --- | --- | --- | --- | --- |
|  | Total | Yes | No | p |
|  | n (%) | | |  |
| Residence |  |  |  | 0.02 |
| Urban | 384 (25.9) | 39 (10.2) | 344 (89.9) |  |
| Rural | 1097 (74.1) | 165 (15.1) | 932 (84.9) |  |
